# Statistical analysis plan for the phaco TIp position during clear corneal Phacoemulsification Surgery (TIPS) randomized controlled trial

**DOI:** 10.1186/s13063-024-07979-0

**Published:** 2024-02-22

**Authors:** Soujanya Kaup, Dimple Kondal, Siddharudha Shivalli, John Buchan

**Affiliations:** 1 Department of Ophthalmology, Yenepoya Medical College Hospital, Yenepoya Deemed to be University, Mangalore, Karnataka 575018 India; 2 DBT/Wellcome Trust India Alliance (Early Career - Clinical and Public Health) Fellow, Hyderabad, 500034 India; 3 Centre for Chronic Disease Control, Safdarjung Development Area, C-1/52, Second Floor, Delhi, 110016 India; 4 Hubert Department of Global Health, Rollins School of Public Health, Emory University, Atlanta, GA USA; 5 Department of Medical Statistics, London School of Hygiene and Tropical Medicine, Keppel Street, London, WC1E 7HT UK; 6 International Centre for Eye Health, London School of Hygiene and Tropical Medicine, Keppel Street, London, WC1E 7HT UK

**Keywords:** Endothelial cell loss, Phacoemulsification, Phacoemulsification/complication, Pseudophakic bullous keratopathy, Phaco-tip, Bevel-up, Bevel-down, Specular microscopy

## Abstract

**Background:**

Cornea is the most important refractive media in the eye, and damage to the corneal endothelium is one of the most common causes of poor visual outcome following cataract surgery, particularly in those with predisposing factors. The role of phaco tip position during phacoemulsification on corneal endothelial damage is ambiguous, and there is no consensus regarding the most cornea-friendly phaco tip position (bevel-up or bevel-down). The objective of the trial is to compare the effect of phaco tip position (bevel-up vs. bevel-down) during phacoemulsification using direct chop technique on corneal endothelial cell count.

**Methods and design:**

TIPS is a randomised, multicentre, parallel-group, triple-masked (participant, outcome assessor, and statistician) trial with 1:1 allocation ratio. A total of 480 eligible participants, aged > 18 years with immature cataract, will be randomly allocated into bevel-up and bevel-down groups at two centres. Randomisation will be stratified according to the cataract grade. The primary outcome is postoperative endothelial cell count at 1 month. Secondary outcomes are central corneal thickness on postoperative days 1, 15, and 30 and difference in intraoperative complications.

**Conclusion:**

In this paper, we describe the detailed statistical analysis plan (SAP) for the TIPS trial, which was prepared prior to database lock. The SAP includes details of planned analyses and unpopulated tables, which will be reported in the publications. We plan to lock the database in July 2023 and publish the results later in the same year.

SAP Version 0.1 (dated: 28 April 2023)

Protocol version:2.0

**Trial registration:**

Clinical Trial Registry of India CTRI/2019/02/017464. Registered on 5 February 2019; https://ctri.nic.in/Clinicaltrials/pmaindet2.php?trialid=29764&EncHid=&userName=2019/02/017464

**Supplementary Information:**

The online version contains supplementary material available at 10.1186/s13063-024-07979-0.

## Background

Globally, the number of cataract surgeries is likely to increase due to increasing ageing population and reduced visual impairment threshold for cataract surgery [1–4]. One can expect an increase in corneal endothelial decompensation, which is one of the most common causes of poor visual outcome following cataract surgery, particularly in those with predisposing factors. However, the impact of phaco tip position during phacoemulsification on corneal endothelial damage is ambiguous, and there is no consensus regarding the most cornea-friendly phaco tip position (bevel-up or bevel-down). Previously, studies investigating the impact of phaco tip position on the endothelium have reported contradictory results and were underpowered [5–7].

The objective of the trial is to compare the effect of phaco tip position (bevel-up vs. bevel-down) on corneal endothelial cell count during phacoemulsification using direct chop phaco-technique.

This document describes the detailed set of statistical analyses to be performed on the data produced by the TIPS trial, to conform with the trial’s protocol. Details on the TIPS design can be found in its protocol [8]. Here, we replicate the main characteristics relevant to understand the data analysis strategy chosen. The document was finalised before database lock and unblinding.

## Study methods

### Trial design

TIPS is a randomised, multicentre, parallel-group, triple-masked (participant, outcome assessor, and statistician) trial with 1:1 allocation ratio. The trial was prospectively registered in the Clinical Trial Registry of India (CTRI/2019/02/017464; registered on 5 February 2019) with all items from the World Health Organization Trial Registration Data Set. This is trial protocol version 2.0 and is published [8].

### Study population

**Fig. 1:**
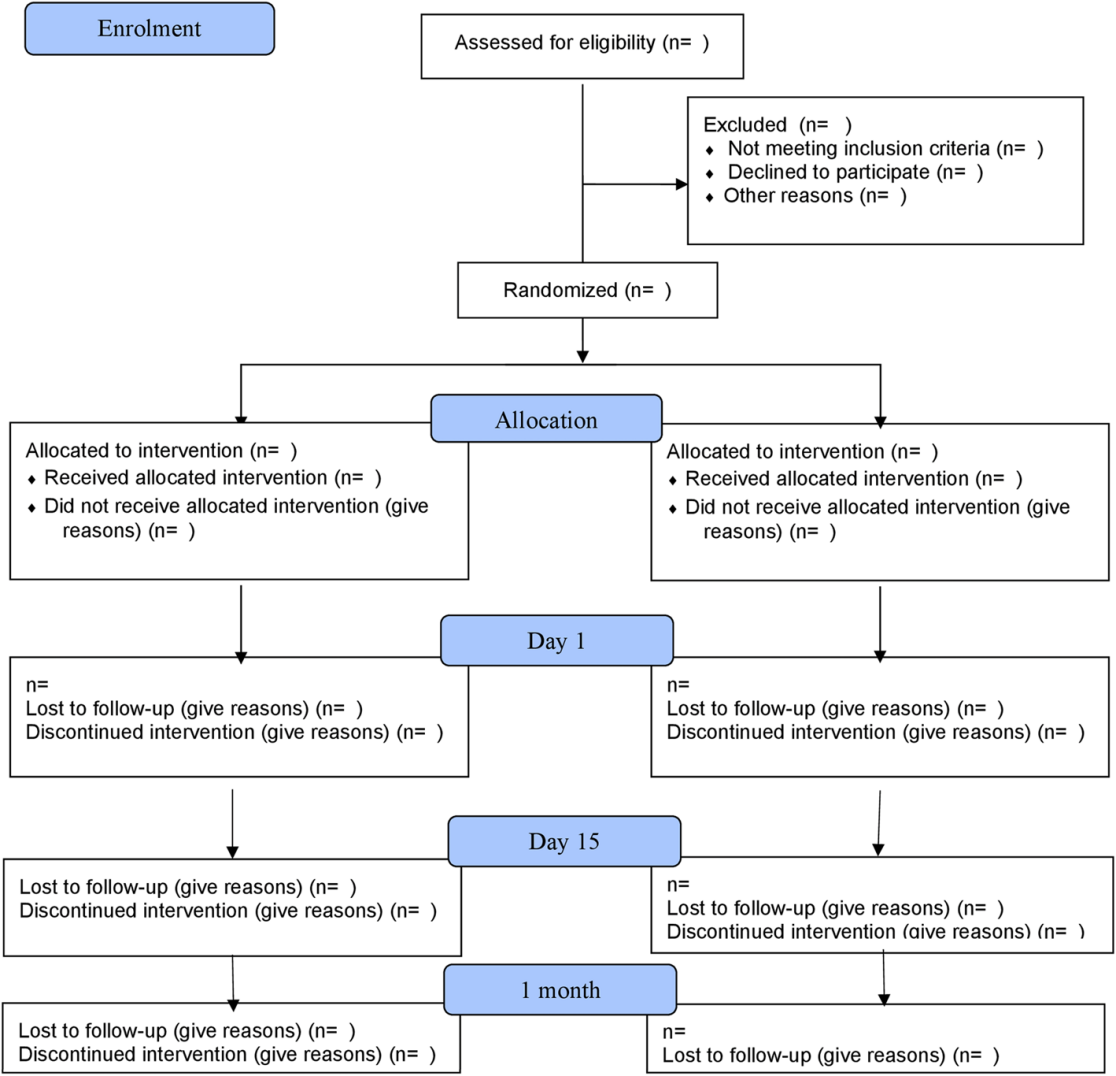
CONSORT (Consolidated Standards of Reporting Trials) flow diagram depicting the progress through the phases of the randomised trial of two groups (enrolment, intervention allocation, follow-up, and data analysis)

The study population consisted of patients from two hospitals in India (Fig. 1), i.e. (1) Department of Ophthalmology, Yenepoya Medical College Hospital, Yenepoya (Deemed to be) University, Mangalore, India, and (2) Netrajyothi Charitable Trust Hospital, Udupi, India.

We included the patients if they met all the inclusion criteria and none of the exclusion criteria as listed in the study protocol and published previously [8].

### Randomisation and masking

**Fig. 2:**
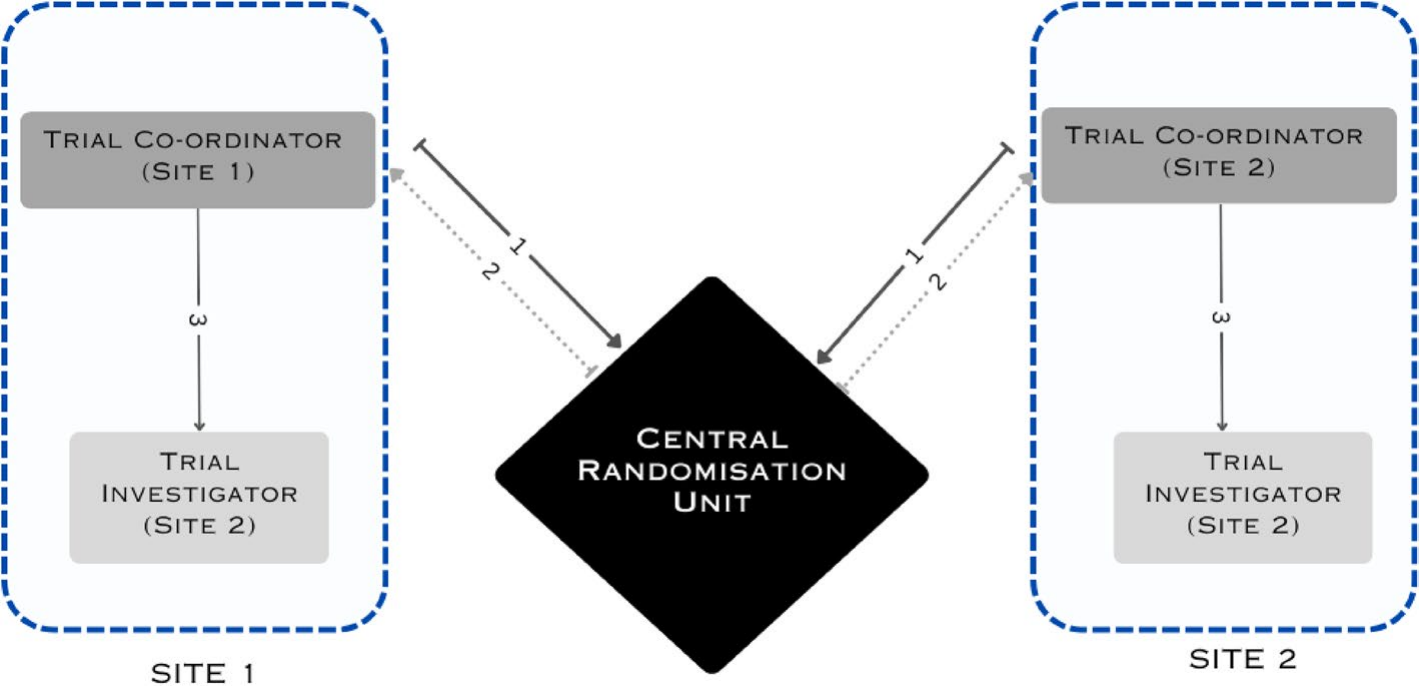
Details of interventions published in the protocol

SS generated a random number sequence using a computer, which was be stored in secured envelopes. Central randomisation with stratified blocks of variable size was used. On the day of surgery, the trial site coordinator will contact the central randomisation unit, and SS will allocate the participants into either of the two groups, i.e. bevel-up or bevel-down. SS will not be in direct contact with the participants and was not involved in participant recruitment. The trial coordinator will inform the surgeon of the trial group after the surgery has begun and capsulorrhexis step has been performed, but before the nucleus emulsification phase (Fig. 2). Stratification was done according to the Lens Opacities Classification System (LOCS) III grading of the cataract into two strata (strata 1: grades 1 and 2 and strata 2: grades 3 and 4). On the day of surgery, the study investigators contacted the central randomisation unit, and SS allocated the participants into either of the two groups, i.e. bevel-up or bevel-down. SS was not in direct contact with the participants. The trial participant, outcome assessor, and statistician were masked. The participants were not aware of the group to which they were randomised and would not be able to differentiate the interventions. The outcome was assessed by a trained research assistant, who was unaware of the intervention group. An independent statistician (DK), who is unaware of the random allocation, will analyse the data.

### Intervention

All surgical interventions will be performed by three surgeons in site one and two surgeons in another site. Details of interventions published in the protocol and Fig. 2 of the published protocol show the steps of the surgery [8].

### Sample size

A sample size of 240 in each group (480 total) was calculated based on a pooled standard deviation of 441.7 (***σ***), for detecting a true mean difference of 138 cells/mm^2^ [2516 (μ1)–2378 (μ2)] [7], to achieve a power of 90% and a level of significance of 5% (two-sided) using the formula below (for two independent samples and continuous outcomes), and adjusting for expected 10% attrition.

$$n=2{\left[\raisebox{1ex}{$\left[Z\left(1-\frac{\upalpha}{2}\right)+Z\left(1-\upbeta \right)\right]$}\!\left/ \!\raisebox{-1ex}{$ ES$}\right.\right]}^2,\textrm{where}\ \textrm{effect}\ \textrm{size},\textrm{ES}=\frac{\left|\upmu 1-\upmu 2\right|}{\upsigma}$$

### Statistical interim analysis and stopping guidance

The data safety and monitoring committee scheduled to review thrice (end of first 50 cases, 160 and 320 cases) and planned to stop or modify the trial for safety purpose if there were more than five serious adverse events in either arm. Serious adverse event is defined as any cause leading to permanent loss of vision of less than 3/60 (best corrected vision) due to the trial intervention (excluding those due to pre-existing conditions).

### Timing of outcome assessments and final analysis

The specific trial outcomes will be measured on postoperative days 1, 15, and 30. The final analysis will be conducted after final data collection, double data entry, data cleaning, and data locking in July 2023.

## Statistical principals

### Trial population

All participants screened for eligibility in this trial will be accounted for, and a CONSORT statement will be prepared (http://www.consort-statement.org) [9] as shown in Fig. 1. The reasons for early withdrawal will be listed for all participants. The number of participants who were screened and found to be eligible but not randomised will be presented, and the reasons for non-participation (where available) will be recorded. Timing and reasons for those lost to follow-up will be presented.

### Confidence intervals and *P* values

Statistical significance level for all outcomes will be at the 0.05 (alpha = 0.05) level. 95% confidence intervals will be presented for all effect estimates.

### Adherence and protocol deviations

The intervention is a one-time surgery; hence, the issues related to adherence by the participants will not occur. The surgeons performing the intervention have been trained to follow the surgical protocol. Adherence to the surgical protocol by the surgeons will be evaluated by random assessment of a sample of surgical video by an independent ophthalmologist who will assign the videos to the treatment groups based on the video assessment. This report will be cross-checked with the original group assignment. The percentage of the verified videos that concur with the original group allocation will be reported. This percentage will give an estimate of the surgical protocol adherence.

Any protocol deviations such as number of cases where the assigned surgical techniques were not followed, where direct chop was not possible, and where surgical technique was modified due to intra-operative complications will be reported.

### Analysis populations

Data will be analysed by the trial statistician who will be blind to random allocation. The analyses will be performed on the principle of ‘intention to treat’ (i.e. will compare patients in the groups to which they were originally randomly assigned). A ‘per-protocol’ analysis will also be performed to analyse the effect of the phaco-tip position on the participants who did not have any major intra-operative complication (posterior capsular rent ± vitreous loss, zonular dehiscence/dialysis, Descemet’s membrane detachment).

### Statistical analysis

All the key trial results will be presented in the tables as shown in Additional file 1.

### Outcome definitions

The primary outcome is as follows: differences in endothelial cell count at 1 month postoperatively between the two groups (i.e. bevel-up and bevel-down group). The principles for use of specular microscope in clinical trials as proposed by McCarey et al. will be followed [10].

#### Secondary outcomes

- Difference between the two groups (i.e. bevel-up and bevel-down group) in central corneal thickness (CCT) on days 1, 15, and 30
- Difference in intraoperative complications by two group (i.e. bevel-up and bevel-down group)

#### Exploratory outcomes

The outcome will also be reported as the following:

- The mean absolute reduction in the endothelial cells between the two groups

Note: Difference in endothelial cells will be calculated as preoperative cell count minus postoperative cell count

- Mean difference in the endothelial cell loss percentage between the two groups

Note: The percentage of endothelial cell loss will also be calculated as = [(preoperative cell count − postoperative cell count)/(preoperative cell count)] × 100%

These exploratory outcomes are also planned to compare our results with previous publications which represent these as their outcomes. Also, these outcomes are more easily understood by practising ophthalmologists as many scientific papers report these outcomes with regard to endothelial cell loss.

#### Baseline comparisons

Baseline descriptive variables of participants will be summarised by treatment group using suitable measures of central tendencies for continuous data (means and medians), variability (SD and interquartile range (IQR)), and frequencies/percentages for categorical data. No significance testing will be undertaken to compare distributional statistics between arms (Additional file 1: Table S1 and S2).

Intra-operative phaco-parameters include if direct chop phacoemulsification technique was used or not, the mean duration (in seconds) and power (%) of ultrasound energy used, the mean amount of irrigating fluid (ml), and the percentage of complications in each group (Additional file 1: Table S3).

#### Primary outcome analysis

The mean endothelial cell counts at 1 month will be compared between the study groups using analysis of covariance (ANCOVA) adjusting for the baseline preoperative endothelial cell counts values (Additional file 1: Table S4). We have considered the ANCOVA approach to adjust for the baseline values and to provide an unbiased estimate of the mean group difference in postoperative endothelial cell count at one month. ANCOVA is an extension of the methods of analysis of variance (ANOVA) and simply requires that we take additional measurements that are statistically related to the response measurements but which are themselves unaffected by the treatments being studied.

The mean absolute reduction in the endothelial cells between the two groups will be compared using ***t***-test or rank sum test after checking the distribution of the outcome. The difference in endothelial cells will be calculated as preoperative cell count minus postoperative cell count. The difference with 95% CI will be reported.

Also, the mean difference in the endothelial cell loss percentage between the two groups will be compared using ***t***-test after checking the distribution of the outcome. The distribution of the outcome will be assessed using Kolmogorov-Smirnov test and Shapiro-Wilk test. The percentage of endothelial cell loss will also be calculated as = [(preoperative cell count − postoperative cell count)/(preoperative cell count)] × 100%.

#### Secondary outcome analysis

The mean difference in CCT on days 1, 15, and 30 between the two groups will be assessed using mixed effect models to account for the repeated measurements from all individuals. The model will include all values collected at days 1, 15, and 30. The model will include treatment group, time (days 1, 15, and 30) as categorical variable and preoperative CCT value, and a random component on the within-individual standard deviation. The interaction between treatment group and time will also be assessed (Additional file 1: Table S5 and S6).

#### Safety endpoint analysis

All adverse events and serious adverse event data collected will be reported for the study population by treatment group (Additional file 1: Table S7).

#### Examination of subgroups (Additional file 1: Table S8)

We will estimate the effect of the intervention by prespecified sub-groups. The heterogeneity of treatment effects will be done by adding interaction terms to the respective models. The variables included in sub-group analysis will be as follows:

- Hardness of cataract (soft vs hard)
- Pre-operative endothelial count (< 2000 cells/mm^3^ vs ≥ 2000 cells/mm^3^)

### Per-protocol analysis and sensitivity analysis

Per-protocol analysis will also be performed excluding patients who experience intraoperative and postoperative complications, as the complications themselves can have a direct impact on the endothelial count.

The sensitivity analysis will be conducted by imputing the missing values for the primary endpoint (that are assumed to be missing at random) using multiple imputations for chained questions [11]. We will generate ten imputed data sets with a maximum number of 1000 iterations, with linear imputation for continuous variables and logistic or multinomial regression for categorical variables. The number of imputations that would be needed will be based on the formula via von Hippel, Paul [12, 13]. The proposed variables that will be included in the imputation model are treatment group, age, sex, preoperative endothelial cell counts, and CCT which are based on the strategy to include at least all variables involved in the planned analysis. In addition, variables not used in the analysis yet have a strong correlation with incomplete variables might be included. The multiple imputation will be performed for the primary outcome only.

### Software details

The statistical analyses will be performed using STATA Version 16.0.

## Conclusion

In this paper, we describe the detailed statistical analysis plan (SAP) for the TIPS trial, which was prepared prior to database lock. All statistical analyses will be conducted as specified in the statistical analysis plan. The SAP includes details of planned analyses and unpopulated tables, which will be reported in the publications.

### Trial status

At the time of first submission, TIPS trial had completed the recruitment, and the planned final follow-up of the patients will be complete by the end of May 2023. We plan to lock the database in July 2023 and publish the results later in the same year.

### Supplementary Information

**Additional file 1: Table S1.** Baseline characteristics. **Table S2.** Preoperative parameters. **Table S3.** Intra-operative parameters. **Table S4.** Primary outcome. **Table S5.** Secondary outcome. **Table S6.** Changes in the central corneal thickness and visual acuity from preoperative period to immediate post-operative period (up to day 30). **Table S7.** Adverse and serious adverse events (SAE) by treatment group. **Table S8.** Subgroup analysis.

**Additional file 2.**

## Data Availability

Not applicable.

## Abbreviations

ANCOVA: Analysis of covariance
CCT: Central corneal thickness
CI: Confidence interval
CONSORT: Consolidated Standards of Reporting Trials
CTRI: Clinical Trial Registry of India
IQR: Interquartile range
LOCS: Lens Opacities Classification System
SAP: Statistical analysis plan
SD: Standard deviation
TIPS: phaco TIp position during clear corneal Phacoemulsification Surgery
