# Supplementary material for: Statistical analysis plan for the phaco TIp position during clear corneal Phacoemulsification Surgery (TIPS) randomized controlled trial
Source: Trials. 2024 Feb 22;25:138. doi: 10.1186/s13063-024-07979-0 (PMC10882898; doi:10.1186/s13063-024-07979-0)
Supplement: Supplementary file 1 — Additional file 1: Table S1. Baseline characteristics. Table S2. Preoperative parameters. Table S3. Intra-operative parameters. Table S4. Primary outcome. Table S5. Secondary outcome. Table S6. Changes in the central corneal thickness and visual acuity from preoperative period to immediate post-operative period (up to day 30). Table S7. Adverse and serious adverse events (SAE) by treatment group. Table S8. Subgroup analysis. [file 13063_2024_7979_MOESM1_ESM.docx]

# Table 1: Baseline characteristics

|  | Overall | Group Bevel-up | Bevel-down |
| --- | --- | --- | --- |
| Age, mean(SD) |  |  |  |
| Sex, n(%) |  |  |  |
| Male |  |  |  |
| Female |  |  |  |
| Diabetes status, n(%) |  |  |  |
| Hypertension status, n(%) |  |  |  |
| Bevel group, n(%) |  |  |  |

# Table 2: Preoperative parameters

|  | Overall | Bevel-up | Bevel-down |
| --- | --- | --- | --- |
| Preoperative Intra Ocular Pressure (mmHg), mean (SD) |  |  |  |
| Preoperative Uncorrected distance visual acuity (LogMAR), mean (SD) |  |  |  |
| Preoperative Corrected distance visual acuity (LogMAR), mean (SD) |  |  |  |
| Grade of cataract, n(%) |  |  |  |
| Grade 1 |  |  |  |
| Grade 2 |  |  |  |
| Grade 3 |  |  |  |
| Grade 4 |  |  |  |
| Preoperative Central corneal thickness (µ), mean (SD) |  |  |  |
| Axial length (mm), mean (SD) |  |  |  |
| Anterior chamber depth (mm), mean (SD) |  |  |  |
| Lens thickness (mm), mean (SD) |  |  |  |
| Maximum pupillary diameter (mm), mean (SD) |  |  |  |
| Preop Specular Endothelial Count (cell/mm^2^), mean (SD) |  |  |  |
| Preop Specular Coefficient of variation (µ), mean (SD) |  |  |  |
| Preop Specular Hexagonal cell percentage (%),mean (SD) |  |  |  |

# Table 3: Intra-operative parameters

|  | Overall | Bevel-up | Bevel-down | p-value |
| --- | --- | --- | --- | --- |
| Direct Chop, n(%) | |  |  |  |
| Yes |  |  |  |  |
| No |  |  |  |  |
| UST*, in sec, mean (SD) | |  |  |  |
| EPT^#^, in sec, mean (SD) | |  |  |  |
| Power (%) |  |  |  |  |
| Irrigating fluid (ml), mean (SD) | |  |  |  |
| Complications, n(%) | |  |  |  |
| Yes |  |  |  |  |
| No |  |  |  |  |

*Ultrasound time #Effective phaco time

# Table 4: Primary outcome

|  | Bevel-up | Bevel-down | Difference (95% CI) | p value |
| --- | --- | --- | --- | --- |
| Endothelial Count (cells/mm^2^) |  |  |  |  |
| Mean absolute reduction in Endothelial Count |  |  |  |  |
| Endothelial cell loss percentage* |  |  |  |  |

*Percentage of endothelial cell loss will also be calculated as = [(preoperative cell count − postoperative cell count)/(preoperative cell count)] × 100%*

# Table 5: Secondary outcome

|  | Bevel-up | Bevel-down | Difference (95% CI) | p value |
| --- | --- | --- | --- | --- |
| Central Corneal Thickness (µ)  Day 1 |  |  |  |  |
| Day 15 |  |  |  |  |
| Day 30 |  |  |  |  |
| Overall Difference |  |  |  |  |

# Table 6: Changes in the central corneal thickness and visual acuity from preoperative period to immediate post-operative period (up to day30)

|  | Bevel-up | Bevel-down |  |
| --- | --- | --- | --- |
| Uncorrected distance visual acuity |  |  |  |
| Preoperative |  |  |  |
| Day 1 |  |  |  |
| Day 15 |  |  |  |
| Day 30 |  |  |  |
| Corrected distance visual acuity |  |  |  |
| Preoperative |  |  |  |
| Day 1 |  |  |  |
| Day 15 |  |  |  |
| Day 30 |  |  |  |
| Central corneal thickness |  |  |  |
| Preoperative |  |  |  |
| Day 1 |  |  |  |
| Day 15 |  |  |  |
| Day 30 |  |  |  |

# **Table 7: Adverse and serious adverse events (SAE) by treatment group**

| Adverse events | Bevel-up | Bevel-down |  |
| --- | --- | --- | --- |
|  |  |  |  |
| SAE^#^ | Bevel-up | Bevel-down |  |
|  |  |  |  |
|  |  |  |  |
|  |  |  |  |

^#^serious adverse events

# **Table 8: Subgroup analysis**

|  |  | Bevel-up | Bevel-down | Difference (95% CI) | p-value for interaction |
| --- | --- | --- | --- | --- | --- |
| Cataract grade | Soft |  |  |  |  |
|  | Hard |  |  |  |  |
| Preoperative endothelial counts | <2000  (cells/mm^2^) |  |  |  |  |
|  | ≥2000 (cells/mm^2^) |  |  |  |  |
